# Exercise prescription for patients with multiple sclerosis; potential benefits and practical recommendations

**DOI:** 10.1186/s12883-017-0960-9

**Published:** 2017-09-16

**Authors:** Farzin Halabchi, Zahra Alizadeh, Mohammad Ali Sahraian, Maryam Abolhasani

**Affiliations:** 1 Sports and Exercise Medicine, Sports Medicine Research Center, Neuroscience Institute, Tehran University of Medical Sciences, Tehran, Iran; 2 Neurology, MS fellowship, MS Research Center, Neuroscience Institute, Tehran University of Medical Sciences, Tehran, Iran; 3 Sports and Exercise Medicine, MS Research Center, Neuroscience Institute, Tehran University of Medical Sciences, Tehran, Iran; 4 Sports and Exercise medicine, Sina MS Research Center, Department of Sports Medicine, Sina Hospital, Hassan Abad Square, Tehran, Iran

**Keywords:** Multiple sclerosis, Exercise, Fitness, Balance, Fatigue

## Abstract

**Background:**

Multiple sclerosis (MS) can result in significant mental and physical symptoms, specially muscle weakness, abnormal walking mechanics, balance problems, spasticity, fatigue, cognitive impairment and depression. Patients with MS frequently decrease physical activity due to the fear from worsening the symptoms and this can result in reconditioning.

Physicians now believe that regular exercise training is a potential solution for limiting the reconditioning process and achieving an optimal level of patient activities, functions and many physical and mental symptoms without any concern about triggering the onset or exacerbation of disease symptoms or relapse.

**Main body:**

Appropriate exercise can cause noteworthy and important improvements in different areas of cardio respiratory fitness (Aerobic fitness), muscle strength, flexibility, balance, fatigue, cognition, quality of life and respiratory function in MS patients.

Aerobic exercise training with low to moderate intensity can result in the improvement of aerobic fitness and reduction of fatigue in MS patients affected by mild or moderate disability.

MS patients can positively adapt to resistance training which may result in improved fatigue and ambulation.

Flexibility exercises such as stretching the muscles may diminish spasticity and prevent future painful contractions. Balance exercises have beneficial effects on fall rates and better balance.

Some general guidelines exist for exercise recommendation in the MS population.

The individualized exercise program should be designed to address a patient’s chief complaint, improve strength, endurance, balance, coordination, fatigue and so on.

An exercise staircase model has been proposed for exercise prescription and progression for a broad spectrum of MS patients.

**Conclusion:**

Exercise should be considered as a safe and effective means of rehabilitation in MS patients. Existing evidence shows that a supervised and individualized exercise program may improve fitness, functional capacity and quality of life as well as modifiable impairments in MS patients.

## Background

MS or demyelinating disease of central nervous system is characterized with neurodegeneration, inflammation, axonal demyelination and transaction [1–3].

This disease has a chronic nature and affects young people, especially women [3]. However, it can be identified in childhood or late adulthood, although this is rare [4–6].

The chronic course of multiple sclerosis can result in significant mental and physical symptoms and irreversible neurologic deficits, including muscle weakness, ataxia, tremor, spasticity, paralysis, balance disorder, cognitive impairment, loss of vision, double vision, vertigo, impaired swallowing and speech, sensory deficits, bladder and bowel dysfunction, pain, fatigue, and depression [3–5, 7].

Motor dysfunctions in MS patients are frequently due to muscle weakness, abnormal walking mechanics, balance problems, spasticity and fatigue [6, 8, 9].

MS has an unpredictable progressive nature and defects and restrictions of activities may suddenly occur and proceed further than the expected time [10].

It is reported that nearly 50% of multiple sclerosis patients use a an accessory device for moving following 15 years from the beginning of disease [11, 12]. Patients frequently reduce their activities due to their fear of symptoms exacerbation [13]. Limited activities increase disability,unfitness, mobility, quality of life (QOL),gait abnormalitiesand lack of stability and muscle strenght [14, 15].

Impairments related to the disease process itself are irreversible by exercise, but impairments resulting from deconditioning are often reversible with exercise [16]. Furthermore, inactivity places MS patients in raised possibility of comorbid health dependent conditions.

Hypercholesterolemia, hypertension, obesity, type 2 diabetes, cancer, arthritis, osteoporosis, depression, fatigue and death from cardiovascular diseases are the most frequently reported comorbid health -related conditions [13, 16].

These comorbidities in MS have further been connected with a raised possibility of inability development because of reduced aerobic capacity, decreased muscle strength, increased muscle atrophyas well as further neurologic risks (e.g., stroke, etc) [16].

For many years, physicians advised newly diagnosed persons with MS to avoid anyphysical activity and exercise. But now, we believe that regular exercise and training is a possible solution during disease period by limiting the deconditioning process and achieving an optimal level of patient activity, functions and many physical and mental health benefits without any concern about a triggering onset or exacerbation of disease symptoms or relapse [13, 16].

We review in this paper, therapeutic function of physical training in multiple sclerosis. The aim of this narrative review is to emphasize the current documents in exercise recommendation including aerobic, resistance, balance or combined trainingin MS patients, and to provide instructions for the sensible use of the physical modalities. Another aim is to outline the impacts of exercise on MS patients by summarizing the physiologic and health view of multiple sclerosis disease.

## Part I

### Physiological profile of MS patients

MS patients, especially with more severe impairments, may exhibit some differences in their physiological characteristics in comparison tohealthy age-matched people in terms of cardiovascular and muscle physiology [16].

Decreased aerobic capacity and cardiorespiratory fitness, in expression of VO_2_maxor maximal oxygen consumption, among MS patients has been about 30% lower than the healthy controls. Respiratory dysfunction due to respiratory muscle weakness and external causes like muscle defect and tiredness are contributing factors in reducing aerobic fitness [14, 16–18].

Another cardiac factors such as basic heart rate and minimum blood pressure are noted to be increased in multiplesclerosis because of impairments in the autonomic control of cardiovascular function that has been estimated about 7% to 60% among MS patients [13, 19].

Also, decreased muscle force calculated by isokinetic and isometric muscle contractions and endurance,muscle mass in total body and increased muscle atrophy are seen in MS patients [13, 16, 20, 21].

It must be shown that muscle strength defect appears particularly clear in the lower extrimities in comparison to the upper extrimities [8, 13].

Flexibility is another physiological characteristic that has diminished in MS patients specially in those with spasticity [16].

About 80% of MS patients feel high temperature intolerance that may be correlated with temporary exacerbation of clinical manifestations of the MS [22]. This is an important concern about MS and exercise. Physical activity is beneficialand important for people with MS, but it should not causeoverheating symptoms [22, 23].

### Benefits of exercise for MS patients

Appropriate exercise can lead to significant and important improvements in different areas of cardiorespiratory fitness (Aerobic fitness), muscle strength, flexibility, stability, tiredness, cognition, quality of life and respiratory function. At this section, the details of benefits are described [3].

### Cardiorespiratory fitness

Aerobic training in MS patients is more extensively studied than resistance training**.** During aerobic training, the patients use multiple muscle acts opposite a low burdon with aim of increasing cardiovascular fitness [16].

In summary, aerobic training of low to moderate intensity is effective on cardiovascular fitness, mood and QOL(quality of life) in multiple sclerosis patients with EDSS < 7. This type of exercise is safe and tolerable in many individuals with MS. multiple sclerosis patients are shown to make favorable gains in cardiorespiratory fitness within a short term of exercise (for example, 4 weeks) [18, 24].

Cardiorespiratory exercise training in MS is associated with increased VO_2_ Max or VO_2_peak and working capacity, respiratory function and reduction of fatigue [18, 25].

A number of studies have made better in cardiorespiratory fitness and aerobic capacity in response to exercise interventions. For instance, Rampello et al. (2007) showed that cardiorespiratory training is better than neurorehabilitation in improvement of functional and moving capacity in multiple sclerosis patients with EDSS < 7 [26].

In another study, Swank et al. (2013) showed that structural cardiorespiratory training can cause improvement in quality of life and emotion of multiple sclerosis patients [2].

In addition cardiorespiratory training can can increase aerobic fitness and reduce tiredness in MS patients some degrees of disability [27]. However, it is not clear whether MS patients with sever impairements have similar adaptations to the cardiorespiratory training benefits or not [13].

### Muscle strength and endurance

During strength training, the patients use muscle contractions against a load for increasing muscle strength. Some studies have demonstrated the benefits strenght exercises in MS patients [16, 28].

Increased muscular strength and endurance have also been shown following other exercise interventions in multiple sclerosis patients [18]. Increased strength in lower limbs, could be an important benefit of strnght training in MS. Strength of the lower limb is affected by the disease often previously and to a more range than arms and hands [29, 30].

White et al. (2004) revealed the effects of the strenght exercise on leg strength, moving ability and self-reported fatigue and disability and showed significant improvements in knee extensor and plantar flexor muscle forces and thenwalking performance [8].

MS patients can make good adjustments to strenght trainings in accompanying by improvement in moving capacity and tiredness [30]. Gutierrez.et al. (2005) revealed that strenght training is a good intervention to improve moving and functional capacity in MS patients having moderate disabilities [9]. Surakka et al. (2004) reported that cardiorespiratory and strength training improves tiredness in MS patients with some degree of disabilities and the training type was more achievable in women patients with less disability in comparison to men patients with more disabilities [27]. In general, resistance training with moderate intensity can induce improvements in muscle strength and function among moderately impaired persons with MS. Thistype of exercise is safe and well tolerated in multiple sclerosis [8, 9, 16, 25, 29].

### Bone health

The use of therapeutic corticosteroids and inactivity may both lead to osteoporosis and pathologic fractures in MS patients. Furthermore, the chronic process of disease and inactivity in multiple sclerosis patients can cause loss of muscle and bone mass. Shabas et al. (2000) showed that among 220 women with MS, 82% had corticosteroid’s history of use and 53% had loss of mobility and bone mass [31].

Weight - bearing exercise can slow the loss of muscle and bone mass in MS. For this reason, the resistance training program is recommended for maintaining and developing the muscle and bone mass in the whole of body [18].

### Flexibility

People with multiple sclerosis frequently have limitation in joint motion because of spasticity and prolonged inactivity. Goals of flexibility exercises are to lengthen the muscles, enhance joint range of motion, reduce spasticity, and maintain good posture and balance [16, 18].

Avoidance of spasticity in early stages of disease is very noted. Lenghening the muscles can delay coming aching muscle contractions and spasms. Studies regarding the effects of flexibility exercise on MS are limited, but this type of exercise are recommended. These exercises must be performed by using proprioceptive facilitation techniques and stretching tight muscles in pelvis, chest, leg and hip flexores. For preventing spasticity aggravation, activities like for example indicating the toes during traing must be prevented [6, 13, 16, 18].

### Balance

Impairments of balance, such as difficulty in maintenance of upright posture, are common in MS patients. Swing during silent standing, moving slowly following postural disturnances and inability to maintain the balance are common in multiple sclerosis and may be related to falling [5, 32, 33].

Some articles showed the effects of balance training in stability of MS patients. Improvements in balance assessed by Berg Balance Scale (BBS), are shown following group aquatic and stability training [34, 35].

Cattaneo et al. (2007) studied the effects of stability training on multiplesclerosis patients and demonstrated that stability training is effective to reduce the falling and improve stability [36].

Generally, balance training has small, but statistically significant effect on improving stability and reducing falling risk in MS patients with some degrees of disabilities. There was limited data on patients with severe MS who are not ambulatory [33, 37].

### Tiredness

Tiredness is greatly seen in MS patients and leads to exacerbation of the neurological and other symptoms of MS such as depression, pain, anxiety and cognitive dysfunction [18, 37]. The underlying mechanisms of fatigue are unknown.

Physical inactivity and mental disorders because of MS or comorbidities have been suggested to cause tiredness.

Exercise can cause some changes such as neuroprotection and neuroplasticity, reduction of long-term inactivityand deregulation of hypothalamus-pituitary- adrenal (HPA) axis and then reduction of tiredness in patients [38].

Evidence has revealed that exercise can manage energy and tierdness levels in healthy peoples. Results are much less conclusive with relation to the exercise and tiedness management in MS patients, although several studies provide support for the potential benefits of exercise in these patients [25, 37, 39].

Cardiorespiratory training and neurorehabilitation, energy storage programs and cooling devices and plans have also been shown as good and effective interventions [24, 40, 41]. Petajan et al. showed that regular aerobic exercisecan reduce fatigue in MS patients, and improve both mood and the QOL(quality of life) [42].

Kargarfard et al. (2012), revealed that aquatic training is effective on tierdness and QOL of women with MS [34].

Establishing a safe and effective exercise program may be considered as an important option while planning for treatment of fatigue and should be encouraged [37].

### Quality of life

HRQOL(Health-related quality of life) has diminished in MS patients. The reduced QOL may be related with deterioration of symptoms, walking and cognition in patients [14].

Stuifbergen (2006) studied the positive effects of regular exercises in general health, liveliness and function of patients [43]. The results of several studies on patients with MS confirm the effectiveness of exercise on long period improvement in physical and social function and quality of life [25, 42, 44].

In summary, exercise training can cause prominent and positive effects in QOL of persons with MS [3].

### CNS morphology and imaging findings

Until now, no evidence has been foundabout the effects of exercise training on brain structure in multiple sclerosis disease. Any way, some studies revealed the effects of cardiorespiratory training on volume of brain grey matter volume and unity of white matter tract as well as functional connectivity of the hippocampus and cortex in people with MS [5, 45]. Despite the limited data on exercise performance on the brain structureon, some studies revealed regular cardiorespiratory training work against brain degeneration in relapsing-remitting type of MS and probably is a protective strategy.

Some studies proposed detection of morphological changes with exercise in the CNS of MS patients by imaging techniques. Although, evidence is still not enoughto demonstrate effects of exercise on brain structure in multiple sclerosis [46].

### Implications for practice

The evidence confirms that individuals with MS are less active than healthy individuals [28]. This is important when designing effective exercise programs for both increasing the tendency and adherence to exercise and createing potential beneficial effects.

Despite all the limitations, exercise has beneficial effects on individuals with multiple sclerosis. Furthermore, no side effects from exercise have been seen in most studies [14, 23, 46–48].

## Part II: Exercise recommendations

Growing evidence exists in favour of exercise as an effective treatment for MS patients, and therefore it should be recommended in the rehabilitation process [7, 49].

Some general guidelines exist for exercise prescription in the MS population. In this part, we will discuss the practical points for exercise prescription in MS patients.

### Pre-exercise evaluation

A comprehensive pre-exercise screening should be considered before designing an individualized exercise program. This should be preferably performed by sports medicine physician, physical medicine and rehabilitation physician, exercise physiologist or physical therapist with proper expertise on MS patients [13, 16, 18, 23, 50].

The evaluation should include a thorough physical examination and history, including MS, functional, and exercise histories. A cardiopulmonary function review should also be done [6, 49]. Patients should also be screened for risk factors or presence of cardiovascular, respiratory or metabolic disorders [51].

Some authors have recommended a baseline ECG or submaximal stress test for this review [52]. However, some others do not always find these tests necessary unless individual cardiovascular risk factors and cardiac history mandate further evaluations [23]. In these cases, MS patients, stratified as “high risk” for medical problems during exercise should undergo a supervised exercise test before participating in an exercise routine [51].

Also, some authors recommend fitness evaluations to be used as a baseline for exercise prescription in MS patients. Using these assessments, the physician can formulate an appropriate initial exercise program [53].

After medical clearance was obtained by physician, exercise professionals should use proper fitness tests to estimate the patient’s cardiorespiratory, musculoskeletal fitness, as well as neuromuscular/functional competence [53]. These tests should be selected according to the patient’s tolerance and goals [18, 54, 55]. Fitness tests should be performed according to the guidelines of American College of Sports Medicine [51]. The six minute walk test (6MWT) requiring minimal apparatus, is a valid tool for MS patients, and is applicable for patients who use walkers, canes, and assistive devices [56, 57]. Other proper tests consist of arm, leg, or combined leg and arm cycle ergometry and recumbent stepping.

Table 1 describes suggestions for testing cardiorespiratory and musculoskeletal fitness, as well as neuromuscular/functional competence.

**Table 1:** Recommendations for exercise testing in MS patients [53–55]

Fitness Parameter	Measures	Comments
Aerobic fitness		
6-min walk test It is used to measure improvements and differences in Pre and Post program performances but not to compare them to “healthy individuals.”	Total distance walked, heart rate, RPE^a^, BP. The HR response to exercise may be decreased due to autonomic dysfunction. Therefore, the use of the RPE scale is preferred in these patients.	Using air conditioner for all aerobic testing. Spasticity, lower limb weakness, and paralysis will preclude walking tests in some patients.
Submaximal, upright, or recumbent leg cycle ergometry. Intermittent instead of continuous protocol may be indicated. Increase work rate by 12–25 W per stage.	Workload and steady-state heart rate to predict VO_2_peak; RPE.	Toe clips and foot straps may be necessary in persons with tremors, spasticity, or weakness in the lower extremities. Begin with a warm-up of unloaded pedaling or cranking.
Combination arm/leg cycle ergometry.	Workload and steady-state heart rate to predict VO_2_peak; RPE.	May reduce difficulty in individuals with lower extremity uncoordination Experience.
Arm ergometry—increase work rate 8–12 W per stage.	Workload and steady-state heart rate to predict VO_2_peak;RPE.	Alternative for persons with lower extremity weakness or paralysis.
Muscular Strength/Endurance		
30-s sit-to-stand test These tests are used to measure improvements and differences in pre- and postprogram performance but not to compare them to “healthy individuals.”	Number of times patient comes to a full stand with arms crossing a standard size chair.	A functional measure of lower extremity strength, power, and muscle endurance.
10RM Testing.	Maximal weight lifted for 10 repetitions (reps).	Machines provide test reliability, support, and joint stability. Remind patients to exhale on concentric action and avoid breath holding.
Flexibility		
Modified bench sit and reach test (1 ft on floor and other straight).	Distance reached in hip/trunk flexion.	Administer test with client seated on a table.
Goniometry.	Range of motion.	Focus on flexibility of hamstrings, hip flexors, ankle plantar flexors, shoulder adductors, and internal rotators.
Power/functional		
Timed up and go test.	Time to stand from a chair, walk a 3-m round trip, and sit back down on the same chair.	Results correlate with gait speed, balance, functional level, the ability to go out.
Five-times sit-to-stand test.	Time to stand and sit 5 consecutive times on a standard size chair.	Most useful in patients ≤60 y.

*BP* blood pressure, *RPE* ratings of perceived exertion, *HR* heart rate, *MS* multiple sclerosis; RM, repetition maximum

^a^RPE is a subjective rating scale ranging from six to 20 that gives an indication of the workout intensity level

### Exercise program

The individualized exercise program should be designed to address a patient’s chief complaint or goal—to improve strength, endurance, balance, coordination, fatigue, etc. It should consider a patient’s baseline impairments and capabilities [18, 50]. The prescription should include all the necessary components, such as frequency, duration, intensity, modalities to be used, and precautions to be observed [50].

### Exercise staircase model

An exercise staircase model has been proposed for exercise prescription and progression for a broad spectrum of MS patients [23].

At the base of the staircase is the passive range of motion exercises. This serves as the foundation and is suitable for the most physically and cognitively disabled. These exercises should be done no less than once daily.

The next step up the staircase is the active range of motion exercises. These are proper for less disabled MS individuals and may be carried out with or without gravity eliminationas strength allows. Even when diffused weakness exists, resistance exercises of cautiously chosen muscles, perhaps not more than 2 per limb, may still permit efficient strengthening. In motivated patients with mild MS, focused muscle strengthening with progressive resistive exercises may be effective.

The third and highest step in the staircase is integrated exercises. Integrated exercises use a combination of strength, endurance, flexibility, balance, and coordination exercises [16, 23]. Recent studies have also shown that combined exercisetraining may have advantages, especiallyin reducing fatigue perception, and improving someaspects of QOL [58, 59]. The exact combination of exercises should be individualized according to patient needs and capabilities. Aquatic exercise is a good example of an integrated exercise, simultaneously incorporating endurance, resistance, flexibility and balance components [16, 23].

### Aerobic exercises

In general, aerobic training of low to moderate intensity produced improvements in aerobic capacity and in measures of HRQL, mood, and depression in patients with mild to moderate MS (EDSS < 7). Aerobic training is generally safe and well tolerated in these patients [13, 16]. Individuals with MS have been shown to make favorable gains in cardiorespiratory fitness within a short span of 4 weeks [18, 60].

Bicycle ergometry, arm ergometry, arm-leg ergometry, aquatic exercise, and treadmill walking may all be suggested, although rowing and running are only recommended for MS patients with proper functioning [13, 54, 55, 60–67]. Currently, the use of robot assisted weight supported treadmills has shown promising results in MS patients [68–71]. Exercise frequency of 2–5 weekly sessions is recommended according to the patient’s toleration. It is preferred to set these sessions in non-resistance training days [13, 53–55]. Starting with intensity of 40%–70% of VO_2_max, 60%–80% of maximal heart rate or 40%–60% heart rate reserve is recommended [13, 18, 53, 63, 64]. A rating of percieved exertion (RPE) scale of 11–13 (fairly light to somewhat hard) is another valuable alternative for exercise intensity. As autonomic dysfunction (a common finding in MS patients) may attenuate the HR response to exercise in MS patients, the use of the RPE scale is advised throughout the exercise [13, 53–55].

Depending on the level of patient’s disability, the initial training duration of 10–40 min is suggested. At first, it may be splitted to three 10-min bouts [13, 53–55]. During the first 2–6 months, progression should be attained by increasing the duration or frequency of exercise sessions. After this time, it should be checked to find out whether a higher intensity is tolerable. In this condition, one training session may be replaced with interval training (up to 90% of VO_2_max) [13, 18, 53–55].

### Resistance exercises

It is important that resistance training should be supervized for safety by an experienced staff until the MS patient is contented with the program [13]. Other than safety concern, it has been shown that supervised is more effective than nonsupervised resistance training [13, 72].

In terms of resistance training modalities, the use of weight machines (i.e., closed kinetic chains) is preferred to free weights (i.e., open kinetic chains) for safety, especially in the initial training phase [13]. If weight machines are not practicable, a home based exercise program using elastic bands and/or body weight as resistance should be considered as a substitute. However, it is not easy to achieve the same benefit from this type of exercises, as it can be achieved using weight machines [13, 18].

Training frequency of 2–3 weekly sessions is well tolerated and gives rise to significant progress in patients. Training intensity should be set in the range of 8 to 15 repetition maximum (RM) with 60%–80% of 1RM. Initial starting intensities of approximately 15 RM is suitable [53–55]. This should be gradually increased over several months toward intensities of approximately 8 to 10 RM [8, 18]. Resistance can be securely added by 2% to 5% when 15 repetitions are properly carried out in successive training sessions [8, 18]. However, day-to-day variability in fatigue will likely justify flexibility in the resistance program. The rate of progression should permit for full recovery between exercise sessions to prevent overuse musculoskeletal injuries [13, 18].

The patient should begin with 1 to 3 sets, which can be gradually increased over a few months to 3 to 4 sets of each exercise. Allow rest breaks of 2 to 4 min between sets and exercises [16].

Regarding the number of exercises, a whole-body program including 4 to 10 exercises is suitable. As a general rule regarding the exercise order, large muscle group exercises should be performed before small muscle group exercises, and multiple-joint exercises before single-joint exercises [13, 53, 73]. Prioritize lower extremity over upper extremity exercises. In MS patients, the lower extremity strength deficit is greater than that of the upper extremity [13, 74].

Balance training of agonist/antagonist muscle groups is also necessary. Particular emphasis should be placed on the posterior shoulder girdle, spine, hip and knee extensors, and dorsiflexor muscles [4–6, 9]. However, any contraindications based on individual impairments should be addressed [16, 18].

Sample exercises include shoulder press, seated scapular row, latissimus pull-downs, chest press, knee extensions, seated leg press, seated hamstring curls, biceps curls, seated triceps extensions, seated back extensions and abdominal crunches, and chair sit to stands [53–55].

In terms of precautions, weight lifting in a seated position (as in most weight machines) is preferred to minimize the risk of falling with free weights. If an individual has impaired proprioception or coordination, the exercise should be done under supervision [16, 18]. Also, compared to the endurance exercise, resistance training in heat sensitive patients less frequently cause symptom exacerbations due to increased body temperature [18].

### Flexibility and stretching exercises

Individuals with MS usually have limited range of motion as a result of spasticity and prolonged immobility. Flexibility exercises are recommended to lengthen muscles, offset the effects of spasticity, enhance joint mobility, and improve balance and posture [18]. These exercises should be performed at least daily for 10 to 15 min [18, 75, 76]. Stretching should be done before and after exercise sessions and must involve both upper and lower body muscle groups used in the program. The neck extensors, anterior shoulder girdle, hip flexors, hamstring, hip adductors and plantar flexors should be especially emphasized [53–55]. Spastic muscles must be particularly targeted. Stretches should be slow, gentle, and prolonged. The stretch should be up to the end of the comfort range and held there for 20 to 60 s. Ballistic stretch or bouncing with the stretch is not recommended. Furthermore, stretching should not be painful. Individuals who need assistance with stretching may use a towel, rope, or partner. For immobilized patients with spasticity, passive stretching may be done by an expert therapist. Passive range of motion above the joint of a paralysed area is recommended. Complementary techniques such as deep breathing, light massage and progressive muscle relaxation techniques may also be beneficial. Supervized yoga or tai chi classes may be suitable for doing stretching exercises in higher-functioning MS patients [16, 18, 62].

### Balance and coordination exercises

Particular attention should be paid to include activities for improvement of balance and coordination. In these activities, the MS patient should shift the centre of gravity and respond to external signals. Swiss ball exercise with coordinated movements and bilateral muscle actions may increase coordination and balance, as well. This type of exercise is extremely helpful to increase strength and flexibility, as well. Tai Chi exercises with slow eccentric movements may also be beneficial to maintain balance, strength and range of motion. For patients with insufficient stability or strength to take part in the mentioned activities, coordination and balance drills may be done in shallow pools. In this milieu, the risk of falling or injury due to balance loss is minimised and support of the water will permit the accomplishment of challenging movements, when it is not possible on land. Improvement of posture, flexibility, coordination and muscle tone are potential advantages of water exercise [6].

### Respiratory muscle training

Adaptation of respiratory muscles to training programs can occur similar to skeletal muscles [18].

In a study, Foglio et al. (1994) examined respiratory muscle function and exercise capacity in MS patients. They concluded that in patients, reduction in exercise tolerance may be associated, at least partially, to diminished respiratory muscle strength [77].

O’Kroy et al. (1993) showed that respiratory muscle training enhanced maximal inspiratory and expiratory pressures, controlled breathing exercises and increased respiratory muscle endurance in MS patients. The use of ventilatory resistance training devices may be helpful and increase respiratory muscle strength [78].

### Special precautions

MS patients are especially susceptible to exercise-related fatigue, heat intolerance, and falling [53–55]. Furthermore, some problems such as spasticity, neurologic or cognitive deficits, and urinary incontinence may influence the exercise program. So, special measures should be considered in these cases. A summary of these precautions and safety recommendations are listed in Table 2.

**Table 2:** Special considerations and precautions for exercise prescription in MS patients

Special considerations	Precautions
Fatigue	Schedule resistance training on non-endurance training days [13, 53, 54].
Spasticity	Consider foot and/or hand straps for ergometers. Use machines instead of free weights [53–55].
Heat intolerance and reduced sweating response	Encourage adequate hydration, keep room temperature between 20 and 22 ° C. Using of cooling fans and precooling before aerobic exercise might have positive effects on performance. It is better to plan exercise in the morning when body temperature is at the lowest [53, 54, 93].
Cognitive deficits	Provide written instructions, diagrams, frequent instructions, and verbal cues [53–55, 94]. Exercise tasks should be initially performed with minimal resistance. Individuals with cognitive impairments may require additional supervision during exercise to ensure their safety [18].
Lack of coordination in extremities	Consider using a synchronized upright or recumbent arm/leg ergometer to ensure balance and safety [53–55, 94].
Sensory loss and balance problems	Perform all exercises preferably in a seated position; use machines or elastic bands instead of free weights [53–55, 94].
Higher energy cost of walking (2–3 times greater than age-matched healthy persons)	Adjust workloads to maintain target heart rate and check heart rate regularly [13, 53–55, 94].
Daily variations in symptoms	Provide close exercise supervision and make daily modifications to exercise variables [13, 53–55, 94].
Urinary incontinence /urgency	Ensure adequate hydration, and schedule exercise in close proximity to restrooms [53–55, 94].
Symptom exacerbation	Discontinue exercises and refer the patient to a physician. Resume exercise program. Once symptoms are stable and the patient is medically ready to continue [13, 53–56, 94].

### Fatigue

There are some concerns about the potential effect of exercise on exacerbation of fatigue in MS patients. However, the existing evidence supports the fact that regular exercise training is linked with a small but important reduction in fatigue among persons with MS [39, 63, 79].

Exercise on elliptical machine may result in significant reduction of fatigue among MS patients. So, this type of exercise may be a useful part of MS rehabilitation programs [80]. Aquatic exercise may also successfully improve fatigue of MS patients and may be considered in the rehabilitation of these patients [34].

### Heat intolerance

A common concern with exercise in MS patients is potentially prompting Uhthoff phenomenon. Uhthoff phenomenon is defined as developing transient symptoms such as amblyopia or blurred vision triggered by overheating from exercise [6, 23]. The exact mechanism of Uhthoff phenomenon is not determined. It may be the result of heat-worsened conduction across partially demyelinated axons, fatigue of damaged neuronal pathways with repetitive nerve transmission, [23, 81] or a hormonal factor produced by cooling [16]. Exercise-induced Uhthoff phenomenon should not be considered as a contraindication for exercise [23]. Fortunately, temporary and mild heat stress causes only transient exacerbation of symptoms without apparent remaining impairment after normothermia is achieved [18]. It often settles within an hour or even sooner with rapid cooling [23]. Moreover, it is still more common in MS patients to respond to exercise in heat conditions with just general fatigue rather than Uhthoff phenomenon with focal neurologic deficits [23, 82]. Studies have demonstrated that usual exercise does not considerably increase core body temperature. A study reported a mean rectal temperature change of 0.1̊ C during land-based exercise and - 0.1̊C during water-based exercise [83]. Alternatively, normal thermoregulatory responses (e.g., sweating and peripheral vasodilatation) that preserve a stable core temperature during usual exercise may be impaired in MS patients. In such cases, an increase in core temperature of even less than 1̊ C may trigger heat-related symptoms [16, 23]. The use of cooling devices such as head-vest liquid cooling garment may provide some modest benefits for MS patients [84, 85]. Another study showed the reduced fatigue and improved ambulation for up to 3 h postcooling with the use of either the liquid cooling system or an icepack suit [23, 85, 86]. When engaging in pool-based aquatic exercises, the ideal water temperature for heat sensitive MS patients seems to be between 27 and 29 °C [18, 23, 87, 88]. Temperatures below 27 ° C can paradoxically enhance spasticity [23, 89].

MS patients, especially those who are heat-sensitive, should avoid scheduling exercise sessions in the hottest times of the day or times when they experience greater fatigue. Exercise sessions in the early morning, when there is cooler temperature and lower body temperature, may be more endurable than in the afternoon [18, 90]. Moreover, resistance exercise is more tolerable than endurance exercise for heat sensitive MS patients and should be encouraged to incorporate resistance exercises in their routines [91].

Particularly for individuals with heat sensitivity, several investigators have recommended pre-exercise cooling strategies, such as the use of cooling devices, [6, 18, 50, 92] cold water lower body immersion, [18] or taking a tepid bath 20 to 30 min before (and after) exercise [23]. Individuals should wear light exercise clothing or may even try exercising with a cooling vest. The exercise area temperature should be kept cool through the use of fans or air conditioning [16, 23].

### Risk of falling

Special attention is needed for patients at high risk of falling due to the balance and coordination problems as well as sensory and proprioceptive deficits. These issues should be particularly considered when planning and supervising exercise sessions in MS patients [13, 16].

## Conclusion

Exercise should be considered as a safe and effective means of rehabilitation in MS patients. Existing evidence has shown that a supervised and individualized exercise program can improve physical fitness, functional capacity, quality of life and modifiable impairments in MS patients. There are general guidelines that may be followed for exercise prescription for the MS population. These guidelines should be adapted according to the patient’s needs, abilities and preferences.

## Abbreviations

BP: blood pressure
EDSS: Expanded Disability Status Scale
HR: heart rate
HRQOL: Health related quality of life
MS: Multiple sclerosis
QOL: Quality of life
RM: repetition maximum
RPE: Rate of precieved exhaustion
RPE: ratings of perceived exertion
VO_2_max: maximal oxygen consumption
VO_2_peak: peak oxygen consumption

## References

[1] Pilutti LA, Platta ME, Motl R, Latimer-Cheung AE (2014). The safety of exercise training in multiple sclerosis: a systematic review. J Neurol Sci [review].

[2] Swank C, Thompson M, Medley A (2013). Aerobic exercise in people with multiple sclerosis: its feasibility and secondary benefits. Int J MS Care.

[3] Motl RW, Sandroff BM (2015). Benefits of exercise training in multiple sclerosis. Curr Neurol Neurosci Rep.

[4] Romberg A, Virtanen A, Aunola S, Karppi SL, Karanko H, Ruutiainen J (2004). Exercise capacity, disability and leisure physical activity of subjects with multiple sclerosis. Mult Scler.

[5] Motl RW, Pilutti LA (2012). The benefits of exercise training in multiple sclerosis. Nat Rev Neurol.

[6] Petajan JH, White AT (1999). Recommendations for physical activity in patients with multiple sclerosis. Sports Med.

[7] Sa MJ (2014). Exercise therapy and multiple sclerosis: a systematic review. J Neurol.

[8] White L, McCoy S, Castellano V, Gutierrez G, Stevens J, Walter G (2004). Resistance training improves strength and functional capacity in persons with multiple sclerosis. Mult Scler.

[9] Gutierrez GM, Chow JW, Tillman MD, McCoy SC, Castellano V, White LJ (2005). Resistance training improves gait kinematics in persons with multiple sclerosis. Arch Phys Med Rehabil.

[10] Asano M, Dawes DJ, Arafah A, Moriello C, Mayo NE (2009). What does a structured review of the effectiveness of exercise interventions for persons with multiple sclerosis tell us about the challenges of designing trials?. Mult Scler.

[11] O'Connor P (2002). Key issues in the diagnosis and treatment of multiple sclerosis. An overview Neurology.

[12] Grima DT, Torrance GW, Francis G, Rice G, Rosner AJ, Lafortune L (2000). Cost and health related quality of life consequences of multiple sclerosis. Mult Scler.

[13] Dalgas U, Stenager E, Ingemann-Hansen T (2008). Multiple sclerosis and physical exercise: recommendations for the application of resistance-, endurance- and combined training. Mult Scler.

[14] Gallien P, Nicolas B, Robineau S, Petrilli S, Houedakor J, Durufle A (2007). Physical training and multiple sclerosis. Ann Readapt Med Phys.

[15] Pilutti LA, Platta ME, Motl RW, Latimer-Cheung AE (2014). The safety of exercise training in multiple sclerosis: a systematic review. J Neurol Sci.

[16] Sandoval AE (2013). Exercise in multiple sclerosis. Phys Med Rehabil Clin N Am.

[17] Feltham MG, Collett J, Izadi H, Wade DT, Morris MG, Meaney AJ (2013). Cardiovascular adaptation in people with multiple sclerosis following a twelve week exercise programme suggest deconditioning rather than autonomic dysfunction caused by the disease Results from a randomized controlled trial. Eur J Phys Rehabil Med.

[18] White LJ, Dressendorfer RH (2004). Exercise and multiple sclerosis. Sports Med.

[19] Huang M, Jay O, Davis SL (2015). Autonomic dysfunction in multiple sclerosis: implications for exercise. Auton Neurosci.

[20] Formica CA, Cosman F, Nieves J, Herbert J, Lindsay R (1997). Reduced bone mass and fat-free mass in women with multiple sclerosis: effects of ambulatory status and glucocorticoid use. Calcif Tissue Int.

[21] Lambert CP, Lee Archer R, Evans WJ (2002). Body composition in ambulatory women with multiplesclerosis. Arch Phys Med Rehabil.

[22] White AT, Wilson TE, Davis SL, Petajan JH (2000). Effect of precooling on physical performance in multiple sclerosis. Mult Scler.

[23] Brown TR, Kraft GH (2005). Exercise and rehabilitation for individuals with multiple sclerosis. Phys Med Rehabil Clin N Am.

[24] Mostert S, Kesselring J. Effects of a short term exercise training programme on aerobic fitness, fatigue, health perception and activity level of subjects with multiple sclerosis. multiple sclerosis. 2002;8(2):161–8. 10.1191/1352458502ms779oa 11990874

[25] Latimer-Cheung AE, Pilutti LA, Hicks AL, Martin Ginis KA, Fenuta AM, MacKibbon KA (2013). Effects of exercise training on fitness, mobility, fatigue, and health-related quality of life among adults with multiple sclerosis: a systematic review to inform guideline development. Arch Phys Med Rehabil.

[26] Rampello A, Franceschini M, Piepoli M, Antenucci R, Lenti G, Olivieri D (2007). Effect of aerobic training on walking capacity and maximal exercise tolerance in patients with multiple sclerosis: a randomized crossover controlled study. Phys Ther.

[27] Surakka J, Romberg A, Ruutiainen J, Aunola S, Virtanen A, Karppi SL (2004). Effects of aerobic and strength exercise on motor fatigue in men and women with multiple sclerosis: a randomized controlled trial. Clin Rehabil.

[28] Motl RW, McAuley E, Snook EM (2005). Physical activity and multiple sclerosis: a meta-analysis. Mult Scler.

[29] DeBolt LS, McCubbin JA (2004). The effects of home-based resistance exercise on balance, power, and mobility in adults with multiple sclerosis. Arch Phys Med Rehabil.

[30] Kjølhede T, Vissing K, Dalgas U (2012). Multiple sclerosis and progressive resistance training: a systematic review. Mult Scler J.

[31] Shabas D, Weinreb H (2000). Preventive healthcare in women with interferon beta-1b in MS: results of an open label trial. Neurol- multiple sclerosis. J Womens Health Gend Based Med.

[32] Motl RW, Learmonth YC, Pilutti LA, Gappmaier E, Coote S (2015). Top 10 research questions related to physical activity and multiple sclerosis. Res Q Exerc Sport.

[33] Cameron MH, Lord S (2010). Postural Control in Multiple Sclerosis: Implications for Fall Prevention. Curr Neurol Neurosci Rep.

[34] Kargarfard M, Etemadifar M, Baker P, Mehrabi M, Hayatbakhsh R (2012). Effect of aquatic exercise training on fatigue and health-related quality of life in patients with multiple sclerosis. Arch Phys Med Rehabil.

[35] Rafeeyan Z, Azarbarzin M, Moosa FM, Hasanzadeh A (2010). Effect of aquatic exercise on the multiple sclerosis patients' quality of life. Iran J Nurs Midwifery Res.

[36] Cattaneo D, Jonsdottir J, Zocchi M, Regola A (2007). Effects of balance exercises on people with multiple sclerosis: a pilot study. Clin Rehabil.

[37] Motl RW (2014). Benefits, safety, and prescription of exercise in persons with multiple sclerosis. Expert Rev Neurother.

[38] Heine M, van de Port I, Rietberg MB, van Wegen EE, Kwakkel G (2015). Exercise therapy for fatigue in multiple sclerosis. Cochrane Database Syst Rev.

[39] Andreasen AK, Stenager E, Dalgas U (2011). The effect of exercise therapy on fatigue in multiple sclerosis. Mult Scler.

[40] Karpatkin HI (2005). Multiple sclerosis and exercise: a review of the evidence. Int J MS Care..

[41] Braley TJ, Chervin RD (2010). Fatigue in multiple sclerosis: mechanisms, evaluation, and treatment. Sleep.

[42] Petajan JH, Gappmaier E, White AT, Spencer MK, Mino L, Hicks RW (1996). Impact of aerobictraining on fitness and quality of life in multiple sclerosis. Ann Neurol.

[43] Stuifbergen AK, Blozis SA, Harrison TC, Becker HA (2006). Exercise, functional limitations, and quality of life: a longitudinal study of persons with multiple sclerosis. Arch Phys Med Rehabil.

[44] Dalgas U, Stenager E, Jakobsen J, Petersen T, Hansen HJ, Knudsen C (2010). Fatigue, mood and quality of life improve in MS patients after progressive resistance training. Mult Scler.

[45] Prakash RS, Snook EM, Motl RW, Kramer AF (2010). Aerobic fitness is associated with gray matter volume and white matter integrity in multiple sclerosis. Brain Res.

[46] Doring A, Pfueller CF, Paul F, Dorr J (2011). Exercise in multiple sclerosis -- an integral component of disease management. EPMA J.

[47] Dalgas U, Stenager E (2005). Multiple sclerosis and physical training. Ugeskr Laeger.

[48] Dalgas U, Stenager E (2012). Exercise and disease progression in multiple sclerosis: can exercise slow down the progression of multiple sclerosis?. Ther Adv Neurol Disord.

[49] Rietberg MB, Brooks D, Uitdehaag BM, Kwakkel G (2005). Exercise therapy for multiple sclerosis. Cochrane Database Syst Rev.

[50] Vollmer T, Benedict R, Bennett S (2012). Exercise as prescriptive therapy in multiple sclerosis. Int J MS Care.

[51] Medicine ACoS. ACSM's Guidelines for exercise testing and prescription: Lippincott Williams & Wilkins; 2013. 10.1249/JSR.0b013e31829a68cf 23851406

[52] Taylor R. Rehabilitation of persons with multiple sclerosis. Phys Med Rehabil Phila: WB Saunders. 1996:1105–7.

[53] Ronai P, LaFontaine T, Bollinger L. Exercise guidelines for persons with multiple sclerosis. Strength & Conditioning Journal. 2011;33(1) 30–3

[54] Jackson K, Mulcare J, Myers J, Nieman D (2009). Multiple sclerosis. ACSM’s Resources for clinical exercise physiology.

[55] Jackson K, Mulchare J, Durstine JL, Moore GE, Painter PL, Roberts SO (2009). Multiple sclerosis. ACSM’s Exercise Management for Persons with chronic diseases and disabilities.

[56] Goldman MD, Marrie RA, Cohen JA (2008). Evaluation of the six-minute walk in multiple sclerosis subjects and healthy controls. Mult Scler.

[57] Savci S, Inal-Ince D, Arikan H, Guclu-Gunduz A, Cetisli-Korkmaz N, Armutlu K (2005). Six-minute walk distance as a measure of functional exercise capacity in multiple sclerosis. Disabil Rehabil.

[58] Gobbi E, Carraro A (2016). Effects of a combined aerobic and resistance exercise program in people with multiple sclerosis: a pilot study. Sport Sciences for Health.

[59] Latimer-Cheung AE, Pilutti LA, Hicks AL, Ginis KAM, Fenuta AM, MacKibbon KA, et al. Effects of exercise training on fitness, mobility, fatigue, and health-related quality of life among adults with multiple sclerosis: a systematic review to inform guideline development. Arch of Phys Med Rehabil. 2013;94(9) 1800–28. e3 10.1016/j.apmr.2013.04.020 23669008

[60] Mostert S, Kesselring J (2002). Effects of a short-term exercise training program on aerobic fitness, fatigue, health perception and activity level of subjects with multiple sclerosis. Mult Scler.

[61] Kileff J, Ashburn A (2005). A pilot study of the effect of aerobic exercise on people with moderate disability multiple sclerosis. Clin Rehabil.

[62] Oken BS, Kishiyama S, Zajdel D, Bourdette D, Carlsen J, Haas M (2004). Randomized controlled trial of yoga and exercise in multiple sclerosis. Neurology.

[63] Petajan JH, Gappmaier E, White AT, Spencer MK, Mino L, Hicks RW (1996). Impact of aerobic training on fitness and quality of life in multiple sclerosis. Ann Neurol.

[64] Ponichtera-Mulcare JA, Mathews T, Barrett PJ, Gupta SC (1997). Change in aerobic fitness of patients with multiple sclerosis during a 6-month training program. Res Sports Med: An Int J.

[65] Schulz K-H, Gold SM, Witte J, Bartsch K, Lang UE, Hellweg R (2004). Impact of aerobic training on immune-endocrine parameters, neurotrophic factors, quality of life and coordinative function in multiple sclerosis. J Neurol Sci.

[66] Van den Berg M, Dawes H, Wade D, Newman M, Burridge J, Izadi H (2006). Treadmill training for individuals with multiple sclerosis: a pilot randomised trial. J Neurol Neurosurg Psychiatry.

[67] Newman M, Dawes H, Van den Berg M, Wade D, Burridge J, Izadi H (2007). Can aerobic treadmill training reduce the effort of walking and fatigue in people with multiple sclerosis: a pilot study. Mult Scler.

[68] Lo AC, Triche EW (2008). Improving gait in multiple sclerosis using robot-assisted, body weight supported treadmill training. Neurorehabil Neural Repair.

[69] Giesser B, Beres-Jones J, Budovitch A, Herlihy E, Harkema S (2007). Locomotor training using body weight support on a treadmill improves mobility in persons with multiple sclerosis: a pilot study. Mult Scler.

[70] Pilutti LA, Lelli DA, Paulseth JE, Crome M, Jiang S, Rathbone MP (2011). Effects of 12 weeks of supported treadmill training on functional ability and quality of life in progressive multiple sclerosis: a pilot study. Arch Phys Med Rehabil.

[71] Wier LM, Hatcher MS, Triche EW, Lo AC (2011). Effect of robot-assisted versus conventional body-weight-supported treadmill training on quality of life for people with multiple sclerosis. J Rehabil Res Dev.

[72] Mazzetti SA, Kraemer WJ, Volek JS, Duncan ND, Ratamess NA, Gomez A (2000). The influence of direct supervision of resistance training on strength performance. Med Sci Sports Exerc.

[73] Kraemer WJ, Adams K, Cafarelli E, Dudley GA, Dooly C, Feigenbaum MS (2002). American College of Sports Medicine position stand. Progression models in resistance training for healthy adults. Med Sci Sports Exerc.

[74] Schwid S, Thornton C, Pandya S, Manzur K, Sanjak M, Petrie M (1999). Quantitative assessment of motor fatigue and strength in MS. Neurology.

[75] Durstine JL. American college of sports medicine's exercise management for persons with chronic diseases and disabilities: human kinetics 10%; 2009.

[76] Burks JS, Johnson KP. Multiple sclerosis: diagnosis, medical management, and rehabilitation: diagnosis, medical management, and rehabilitation: demos medical; 2000.

[77] Foglio K, Clini E, Facchetti D, Vitacca M, Marangoni S, Bonomelli M (1994). Respiratory muscle function and exercise capacity in multiple sclerosis. Eur Respir J.

[78] O'Kroy JA, Coast JR (1993). Effects of flow and resistive training on respiratory muscle endurance and strength. Respiration.

[79] Pilutti LA, Greenlee TA, Motl RW, Nickrent MS, Petruzzello SJ (2013). Effects of exercise training on fatigue in multiple sclerosis: a meta-analysis. Psychosom Med.

[80] Huisinga JM, Filipi ML, Stergiou N (2011). Elliptical exercise improves fatigue ratings and quality of life in patients with multiple sclerosis. J Rehabil Res Dev.

[81] Van Diemen H, Van Dongen M, Dammers J, Polman C (1992). Increased visual impairment after exercise (Uhthoff’s phenomenon) in multiple sclerosis: therapeutic possibilities. Eur Neurol.

[82] Ponichtera-Mulcare JA (1993). Exercise and multiple sclerosis. Med Sci Sports Exerc.

[83] Ponichtera-Mulcare J, Glaser R, Mathews T, Camaione D (1993). In the Field-Maximal aerobic exercise in persons with multiple sclerosis. Clinical Kinesiology.

[84] Capello E, Gardella M, Leandri M, Abbruzzese G, Minatel C, Tartaglione A (1995). Lowering body temperature with a cooling suit as symptomatic treatment for thermosensitive multiple sclerosis patients. Ital J Neurol Sci.

[85] Kraft GH, Alquist AD (1997). Effect of microclimate cooling on physical function in multiple sclerosis (MS). J Rehabil Res Dev.

[86] Syndulko K, Woldanski A, Baumhefner RW, Tourtellotte WW (1995). Preliminary evaluation of lowering tympanic temperature for the symptomatic treatment of multiple sclerosis. Neurorehabil Neural Repair.

[87] Peterson C (2001). Exercise in 94 degrees F water for a patient with multiple sclerosis. Phys Ther.

[88] Peterson J, Bell G (1995). Aquatic exercise for individuals with multiple sclerosis. Clinical Kinesiology.

[89] Chiara T, Carlos J, Martin D, Miller R, Nadeau S (1998). Cold effect on oxygen uptake, perceived exertion, and spasticity in patients with multiple sclerosis. Arch Phys Med Rehabil.

[90] Noronha M, Vas C, Aziz H (1968). Autonomic dysfunction (sweating responses) in multiple sclerosis. J Neurol Neurosurg Psychiatry.

[91] Skjerbaek AG, Moller AB, Jensen E, Vissing K, Sorensen H, Nybo L (2013). Heat sensitive persons with multiple sclerosis are more tolerant to resistance exercise than to endurance exercise. Mult Scler.

[92] Grahn DA, Murray JL, Heller HC (2008). Cooling via one hand improves physical performance in heat-sensitive individuals with multiple sclerosis: a preliminary study. BMC Neurol.

[93] Mulchare J, Webb P, Mathews T, Gupta S (2001). Sweat response in persons with multiple sclerosis during submaximal aerobic exercise. Int J Mult Scler Care.

[94] LaFontaine T. Clients with spinal cord injury, multiple sclerosis, epilepsy, and cerebral palsy. NSCA’s Essentials of Personal Training Earle RW and Baechle TR, eds Champaign, IL: Human Kinetics. 2004:565–8.

